# Changes in the endurance shuttle walk test in COPD patients with chronic respiratory failure after pulmonary rehabilitation: the minimal important difference obtained with anchor- and distribution-based method

**DOI:** 10.1186/s12931-015-0182-x

**Published:** 2015-02-19

**Authors:** Wytske A Altenburg, Marieke L Duiverman, Nick HT ten Hacken, Huib AM Kerstjens, Mathieu HG de Greef, Peter J Wijkstra, Johan B Wempe

**Affiliations:** University of Groningen, University Medical Centre Groningen, Department of Pulmonary Diseases, Groningen, the Netherlands; University of Groningen, University Medical Centre Groningen, Centre for Rehabilitation, CD24, PO Box 30002, 9750 RA Haren, The Netherlands; University of Groningen, University Medical Centre Groningen, Institute of Human Movement Sciences, Groningen, the Netherlands; University of Groningen, University Medical Centre Groningen, Groningen Research Institute for Asthma and COPD, Groningen, the Netherlands

**Keywords:** Endurance shuttle walk test, Minimally important difference, COPD, Respiratory failure

## Abstract

**Background:**

Although the endurance shuttle walk test (ESWT) has proven to be responsive to change in exercise capacity after pulmonary rehabilitation (PR) for COPD, the minimally important difference (MID) has not yet been established. We aimed to establish the MID of the ESWT in patients with severe COPD and chronic hypercapnic respiratory failure following PR.

**Methods:**

Data were derived from a randomized controlled trial, investigating the value of noninvasive positive pressure ventilation added to PR. Fifty-five patients with stable COPD, GOLD stage IV, with chronic respiratory failure were included (mean (SD) FEV_1_ 31.1 (12.0) % pred, age 62 (9) y). MID estimates of the ESWT in seconds, percentage and meters change were calculated with anchor based and distribution based methods. Six minute walking distance (6MWD), peak work rate on bicycle ergometry (Wpeak) and Chronic Respiratory Questionnaire (CRQ) were used as anchors and Cohen’s effect size was used as distribution based method.

**Results:**

The estimated MID of the ESWT with the different anchors ranged from 186–199 s, 76–82% and 154–164 m. Using the distribution based method the MID was 144 s, 61% and 137 m.

**Conclusions:**

Estimates of the MID for the ESWT after PR showed only small differences using different anchors in patients with COPD and chronic respiratory failure. Therefore we recommend using a range of 186–199 s, 76–82% or 154–164 m as MID of the ESWT in COPD patients with chronic respiratory failure. Further research in larger populations should elucidate whether this cut-off value is also valid in other COPD populations and with other interventions.

**Trial registration:**

ClinicalTrials.Gov (ID NCT00135538).

**Electronic supplementary material:**

The online version of this article (doi:10.1186/s12931-015-0182-x) contains supplementary material, which is available to authorized users.

## Background

Patients with severe Chronic Obstructive Pulmonary Disease (COPD) have an impaired exercise capacity, which is frequently associated with lower physical activity level [1] and, importantly, with lower quality of life [2,3] and higher mortality [4]. Therefore, enhancement of exercise capacity is an important goal of interventions such as pulmonary rehabilitation (PR) [5] and pharmacological treatment [6].

Exercise capacity can be assessed by maximal and submaximal tests, which reflect different pathobiological properties like sufficient aerobic enzyme systems, muscle strength, cardiovascular fitness and motivation. Maximal exercise capacity is commonly assessed with incremental cycle ergometry or treadmill walking, but these tests are not very responsive to interventions such as medication [7] or exercise training [8]. Field walking tests, such as the six-minute walking distance (6MWD) and endurance shuttle walk test (ESWT), might better reflect quality of life and activities of daily living, and therefore might be more relevant measurements in patients with severe COPD. The ESWT has a number of advantages compared to the 6MWD. Firstly, the ESWT has been shown to be very responsive to bronchodilation [9] and PR [10,11] and might be even more responsive to treatment than the 6MWD [11,12]. Secondly, the ESWT is less affected by motivation and pacing ability of the patient than the 6MWD because gate speed is imposed [13,14].

Unfortunately, at this moment it is not clear which size of change in ESWT could be considered as clinically relevant. This is important for developing new studies, for weighing results of interventions, and for clinicians in evaluating provided care. Until now, only one study provided some information about the minimally important difference (MID) of the ESWT [15]. In that study, the MID was investigated after bronchodilation and PR using an anchor based (global rating of change) and distribution based method (half a standard deviation (SD) of the changes in ESWT time or distance) [15]. The MID of the ESWT after treatment with salmeterol or ipratropium bromide was found to be 45–85 seconds. The anchor based MID of the ESWT after 7 weeks of PR, however, was not determined.

For this study we used the Chronic Respiratory Questionnaire (CRQ), peak work rate (Wpeak) and 6MWD as anchors in combination with a distribution based method. Those anchors were chosen because they are accepted outcomes of pulmonary rehabilitation and all in some way reflect exercise capacity or quality of life. The aim of the current study is to determine the MID of the ESWT in patients with severe, hypercapnic COPD (GOLD IV) following PR with or without nocturnal non-invasive intermittent positive pressure ventilation (NIPPV).

## Methods

### Study population

A total of 55 patients with stable COPD, GOLD stage IV, with chronic hypercapnic respiratory failure, were included in the current analyses. All these patients participated in a randomized controlled trial of which the results were published previously [16,17]. In this study the additional effect of NIPPV on the effects of PR was investigated. Seventy-two patients were included in the study, of which 56 patients completed the rehabilitation program of 12 weeks. Patients were assigned to rehabilitation with (n = 32) or without NIPPV (n = 24). Inclusion criteria were a stable clinical condition (no exacerbation in the four weeks prior to study participation), severe COPD (FEV_1_ < 50%pred), hypercapnia at rest (arterial carbon dioxide pressure PaCO_2_ > 6.0 kPa while breathing room air) and age between 40–80 years. Exclusion criteria were the presence of cardiac or neuromuscular diseases limiting exercise tolerance, exposure to NIPPV or PR in the previous 18 months, or the presence of obstructive sleep apnoea syndrome (apnoea/hypopnoea index ≥ 10/hour). The study was approved by the local Medical Ethics Committee of the University Medical Centre Groningen and was registered at ClinicalTrials.Gov (ID NCT00135538). All patients gave written informed consent to participate. In the current analysis only patients that completed all measurements (31 in the PR group and 24 in the PR + NIPPV group) at the start and directly after the rehabilitation program were included.

### Study design

At baseline lung function, exercise tolerance and quality of life were assessed. Thereafter patients started a 12 week period of rehabilitation with or without NIPPV. The rehabilitation program consisted of strength training, cycling, walking, inspiratory muscle training, education and psychological and/or nutritional support if necessary as described in detail elsewhere [16,17]. The exercise training sessions were on three days per week. NIPPV was initiated in the hospital to train the patients to use the ventilatory support during the night as long as possible with a minimum duration of 6 hours. The adjustment process of NIPPV and the patient compliance afterwards were described in detail in previous publications [16,17]. After the intervention period of 12 weeks measurements of exercise tolerance and quality of life were repeated. Apnea/hypoapnea index was measured with overnight polygraphy prior to the study.

### Measurements

All lung function measurements were performed after bronchodilation. Forced expiration volume in 1 second (FEV_1_) and forced vital capacity (FVC) were assessed by spirometry according to ERS criteria [18]. Total lung capacity (TLC) and residual volume (RV) were measured by body plethysmography [19].

Cycle ergometer tests were performed after optimal bronchodilation. First, daytime resting arterial blood gases on room air were taken from all patients while lying (Rapid lab type 865, Siemens, USA). The incremental cycle ergometer test was performed using a 1-minute incremental protocol, using 5 Watt increments. Patients were seated on the bicycle, respired through a mouth piece and wore a nose clip during the test. Minute ventilation, tidal volume, breathing frequency and oxygen uptake were measured continuously (Oxycon Pro Viasys, Bilthoven, the Netherlands). Maximal workload (Wpeak) was defined as the highest work level reached and maintained for at least 30 seconds.

The ESWT was performed on a 10 m long course at a speed corresponding with 85% of VO_2_ peak, which was estimated from an earlier performed incremental shuttle walking test [20]. For both tests a practice walk was done during the run-in period of the study. Patients were instructed to walk as long as possible at the speed that was dictated by the auditory signal. The test was ended when a patient was more than 0.5 m away from the marker before the signal was given on two successive shuttles, or when he or she indicated to be exhausted.

The 6MWD was assessed indoors on a 40 m long course. Patients used their usual walking aids and, if applicable, their usual ambulatory oxygen therapy during the test. The test assistant gave standardized encouragements every 30 seconds and told the patient after 2 and after 4 minutes that he/she was 2 and 4 minutes on his/her way [21]. All patients performed a practice test first, the results of which were discarded.

During the walking tests supplemental oxygen was permitted, but conditions were the same at all tests. The walking tests were not stopped because of desaturation.

Quality of life was assessed with the interviewed version of the Chronic Respiratory Questionnaire [22]. The CRQ is a widely used disease specific questionnaire which has shown to be reliable and valid in COPD patients [23].

### Minimal important difference calculation methods and statistical analysis

The minimal important difference can be estimated in various ways and generally a combination of methods is recommended [24]. In the current study we used both anchor based and distribution based methods. For the anchor based method we used three measures with an established MID: The 6MWD (25 m) [25], Wpeak (4 Watt) [26,27] and the CRQ (10 points in total score) [28]. Spearman’s correlations between improved ESWT on the one hand and improved 6MWD, Wpeak and CRQ on the other hand had to be > 0.30 allowing linear regression analyses. There is no consensus on what an appreciable association between the outcome variable and the anchor should be. However, a correlation of 0.30 is considered to be the lower limit [24,26,29]. Afterwards, the MID of the ESWT was derived from substituting the MID of the anchors in the regression line of ∆ESWT and ∆ anchor. A distribution-based method was used to compare the change in outcome variable with an arbitrary measure of variability, which in this study was the effect size, using the following equation: 0.5 *SD (ΔESWT). The analyses were performed on pooled treatment groups as the separate analyses of the two treatment arms (PR or PR + NIPPV) showed similar associations, thereby increasing statistical power. The MID of the ESWT was expressed as change in time, percentage change from baseline (time or meters) and change in meters.

All statistical analyses were performed using Scientific Package of Social Sciences (SPSS) version 18.0. P-values <0.05 were considered to be significant.

## Results

The baseline characteristics of the 55 COPD patients (42% female) are shown in Table 1.

**Table 1:** **Baseline characteristics**

	**(n = 55)**
Age, y	62.0 (9)
BMI, kg/m^2^	27.5 (6.1)
FEV_1_,l	0.84 (0.34)
FEV_1_ %pred, %	31.1 (12.0)
FEV_1_/FVC, %	31.8 (9.2)
TLC, l	7.27 (1.41)
RV/TLC, %	63.9 (9.1)
PO_2_, kPa	8.14 (1.18)
PCO_2_, kPa	6.85 (0.73)
Wpeak, W	28.1 (18.5)
VO_2_ peak, ml/min	250 (56)
ISWT, m	160 (40–450)
ESWT, m	240 (40–1160)
ESWT, s	257 (81–1220)
6MWD, m	315 (80–615)
CRQ total	80.3 (17.8)
CRQ dyspnoea	16.7 (4.8)
CRQ fatigue	13.68 (4.5)
CRQ emotion	31.5 (7.9)
CRQ mastery	18.4 (5.0)

Values or expressed as mean (SD) or median (range). BMI: body mass index; FEV_1_: forced expiration volume in 1 second; FVC: forced vital capacity; TLC: total lung capacity; RV: residual volume; Wpeak: peak work rate; ISWT: incremental shuttle walk test; ESWT: endurance shuttle walk test; 6MWD: six minute walking distance; CRQ: Chronic Respiratory Questionnaire.

### Correlates of ESWT change with change in anchors

For further analysis correlations >0.30 were used. These were shown for ESWT change in absolute time (ΔESWTs) and % change from baseline (ΔESWT%) with all three anchors, 6MWD, Wpeak and CRQ, and for ESWT change in meters (ΔESWTm) with the CRQ and Wpeak. (Table 2 and Additional file 1: Figure S1) The CRQ total score showed higher correlations with ESWT change than the CRQ subscales and therefore CRQ total score was used in the regression analysis.

**Table 2:** **Spearman’s Correlation of ESWT with possible anchors**

	**ΔESWT (s)**	**ΔESWT (%)**	**ΔESWT (m)**
∆ Wpeak, W	**0.568**	**0.525**	**0.610**
∆ CRQ total	**0.449**	**0.441**	**0.428**
∆ CRQ dyspnoea	**0.313**	0.299	**0.305**
∆ CRQ fatigue	**0.373**	**0.328**	**0.350**
∆ CRQ emotion	**0.363**	**0.351**	**0.353**
∆ CRQ mastery	**0.389**	**0.338**	**0.381**
∆ 6MWD, m	**0.307**	**0.318**	0.275

Values printed bold p < 0.05.

Wpeak: peak work rate; CRQ: Chronic Respiratory Questionnaire; 6MWD: six minute walking distance; ΔESWT: change in endurance shuttle walking test.

### Anchor based and distribution based estimates of the MID of the ESWT

Table 3 shows the MID estimates and 95% confidence intervals (in seconds, percentage and meters) derived from the linear regression equations of ΔESWT and Δanchor (Additional file 2: Table S1). The MID estimate of ESWTm using the 6MWD as an anchor was not calculated because ΔESWTm and Δ6MWD correlated <0.30. The MID estimates calculated with the distribution based method were 145 s for ΔESWTs, 61.4% for ΔESWT% and 137 m for ΔESWTm.

**Table 3:** **Anchor-based MID of ESWT (s and %) using CRQ and 6MWD**

	**anchor**	**MID ESWT**	**95% CI**
MID ESWT (s)	Wpeak	198.9	163.7–234.1
	CRQ	186.4	147.4–225.4
	6MWD	199.1	153.3–245.0
MID ESWT (%)	Wpeak	81.2	65.3–97.0
	CRQ	75.9	59.2–92.6
	6MWD	82.2	63.5–100.9
MID ESWT (m)	Wpeak	163.6	129.1–198.2
	CRQ	153.7	115.6–191.8

Wpeak: peak work rate; CRQ: Chronic Respiratory Questionnaire; 6MWD: six minute walking distance; ΔESWT: change in endurance shuttle walking test.

## Discussion

In this population of patients with severe COPD and chronic hypercapnic respiratory failure, who followed PR (with or without NIPPV), we estimated the MID for the ESWT. Using three different anchors we found MID estimates ranging from 186–199 s, 76–82% and 154–164 m. The anchors Wpeak and 6MWD provided almost identical results. In contrast, the MID based on statistical variability of the effect size was somewhat lower: 61%, 145 s and 137 m.

All anchors used in our study meet the criteria of a good anchor [24]. These criteria, applied to this field, are firstly: the anchor should have an established MID derived from multiple high quality studies, including many well characterized COPD patients, using multiple methods, and agreeing about the final MID estimate. Secondly, the anchor should be derived from a comparable COPD population. Thirdly, the anchor should somehow reflect the perception of improved exercise capacity. Finally the anchor should correlate highly with changes in ESWT. The anchors used in this study will be discussed in this perspective.

The first anchor was the Chronic Respiratory Questionnaire. The CRQ has a widely accepted MID of 10 points on the total score (or 0.5 per question), which has been estimated with different methods [28,30,31]. In addition, the CRQ has served as an anchor for establishing MID’s of other measurement tools as well, such as the Hospital Anxiety and Depression Scale and the feeling thermometer [32,33]. Correlations of CRQ change and ESWT change showed rho values > 0.40 and the CRQ could therefore be used as an anchor. Although the CRQ does not reflect the exact same concept as the ESWT, it is a measure that reflects the patients perception of health status, which agrees with the MID concept.

The second anchor was Wpeak. Two studies estimated the MID for Wpeak using anchor based or distribution based methods and expert opinions. Both studies suggest a MID of 4 Watt. [26,27] Correlations of Wpeak change and ESWT change showed rho values > 0.50 suggesting that Wpeak could be used as an anchor. In addition, Wpeak reflects exercise capacity as does the ESWT, and in both tests patients reach a similar peak performance [34].

The 6MWD also seems a suitable anchor as its MID has been established in different subgroups of COPD patients using different interventions including a PR intervention. We chose to use the MID of 25 m, based on two studies, one in a slightly milder COPD population (age 70 y, FEV_1_ 52% pred) after PR [25], and one in a more severe COPD population (age 66.4, FEV_1_ 26.9% pred), showing an MID of 26 m after surgical lung volume reduction [26]. Another feature demonstrating the 6MWD to be a suitable anchor is that it reflects exercise capacity, as does the ESWT, though perhaps in a different way. Associations of 6MWD change with ESWT change were just high enough to use it as an anchor, therefore this estimation should be taken with some caution. On the other hand, the MID estimated using the 6MWD as anchor was very similar to the MID estimated using the CRQ and Wpeak.

To verify the MID values found with the anchor based method we also used a distribution based method. The latter method should only be used complementary to an anchor based method as it does not fit with the primary aim of the MID concept, which is to indentify an effect size that is relevant in the perception of the patient [24]. In our study the distribution based method showed lower values for the MID than the anchor based method. It has been shown before that distribution based methods tend to underestimate the MID when based on a single study [32].

The MID values estimated with the CRQ, Wpeak and 6MWD were very close to one another (see Table 3). The MID estimated with the distribution based method was somewhat lower (144 s, 61% and 137 m). In line with the literature we recommend to use MID values based on the anchor based method [24]. We prefer to use the MID estimates in seconds and %, and not meters as a change of 100 meters at a low speed has a different value in clinical perspective than a change of 100 m at a high speed.

The estimation of the MID of the ESWT after PR has been investigated in one other study in patients with less severe COPD (FEV_1_ 48% pred). In this study, global rating of change was used as an anchor, comprising of one question. To our opinion, the anchors in our study were suitable as well because the CRQ, 6MWD and to a lesser degree Wpeak, are outcome measures with an established MID and showed correlations >0.30 with ESWT change. However, we realize that our anchors might not reflect exactly the same construct as the ESWT. Pepin et al. only used a distribution based method showing an MID of 186 seconds or 136%, because they decided that associations with the global rating of change were too low to be used to estimate an anchor based MID after PR.

Though we studied a different population than Pepin, our anchor based MID was surprisingly similar to their distribution based estimate, at least for the change in ESWT (s).

Of course we were very interested in how our results are in comparison to other PR intervention studies that used the ESWT as an outcome measure. Several studies were found and they all showed significant improvements in ESWT after intervention [10,35-39]. The studies included patients with quite similar mean age (60–75 years) and mean FEV_1_ (37.5–63.0% predicted) and demonstrated mean ESWT improvements between 138–378 seconds, 15–100% and 106–393 meters [10,35-39]. These change values were consistent with our MID estimates (ranging from 186–199 s, 76–82% and 154–164 m).

A major difference with the patients included in the present analysis is that the patients in the current analysis all suffered from chronic hypercapnic respiratory failure. Probably, the differences found in mean ESWT changes in these studies are due to differences in COPD population, study design and efficacy of the intervention.

A strong point of our study is that we used an anchor based method including three different anchors as well as a distribution based method to determine the MID of the ESWT. Another strong feature is that the correlations of the ESWT change with all anchors were above 0.30, allowing us to use the anchors in a meaningful way.

A limitation of this study might be the relatively small number of patients in the study and the fact that this was a very specific group of COPD patients, namely those with severe COPD and chronic respiratory failure. Therefore, it might not be appropriate to generalize our results straight to the entire COPD population as the MID might differ between populations with other phenotypes and disease stages. A remark should also be made about the confidence intervals for the MID estimates we found, which were quite wide. This hinders firm conclusions. Another limitation of this study is that we did not use a global rating of change as an anchor to estimate the MID of the ESWT. For example by asking the patient the question: ‘In comparison with your previous test, how would you rate your performance on the current test? Using a 7-point Likert scale to rate this performance.

## Conclusions

In conclusion, in patients with severe hypercapnic COPD undergoing PR with or without NIPPV, the MID estimates for the ESWT ranged from 186–199 s, 76–82% and 154–164 m using different anchors (CRQ, Wpeak, 6MWD). Estimates of the MID of the ESWT using 6MWD and Wpeak as anchors provide almost identical results. This MID construct needs further validation in larger and different subgroups of COPD patients.

## Additional files

Additional file 1: Figure S1.
**a-i**. Scatterplots of change in ESWT versus change in anchor variable. Additional file 2: Table S1. Regression equations used to calculate MID values.

## Abbreviations

COPD: Chronic Obstructive Pulmonary Disease
6MWD: Six minute walking distance
BMI: Body mass index
ESWT: Endurance shuttle walk test
MID: Minimally important difference
PR: Pulmonary rehabilitation
SD: Standard deviation
NIPPV: Non-invasive intermittent positive pressure ventilation
CRQ: Chronic Respiratory Questionnaire
Wpeak: Peak work rate
FEV_1_: Forced expiration volume in one second
FVC: Forced vital capacity
ERS: European Respiratory Society
TLC: Total lung capacity
RV: Residual volume
SPSS: Scientific Package of Social Sciences
∆ESWTs: Change in endurance shuttle walk test in seconds
∆ESWT %: Percentage change in endurance shuttle walk test
∆ESWT m: Change in endurance shuttle walk test in meters
