# Supplementary figures and images for: Changes in the endurance shuttle walk test in COPD patients with chronic respiratory failure after pulmonary rehabilitation: the minimal important difference obtained with anchor- and distribution-based method
Source: Respir Res. 2015 Feb 19;16(1):27. doi: 10.1186/s12931-015-0182-x (PMC4336738; doi:10.1186/s12931-015-0182-x)

f

e

d

c

b

a

rho=0.428

rho=0.441

rho=0.449

rho=0.610

rho=0.525

rho=0.568

i

h

g

rho=0.275

rho=0.318

rho=0.307


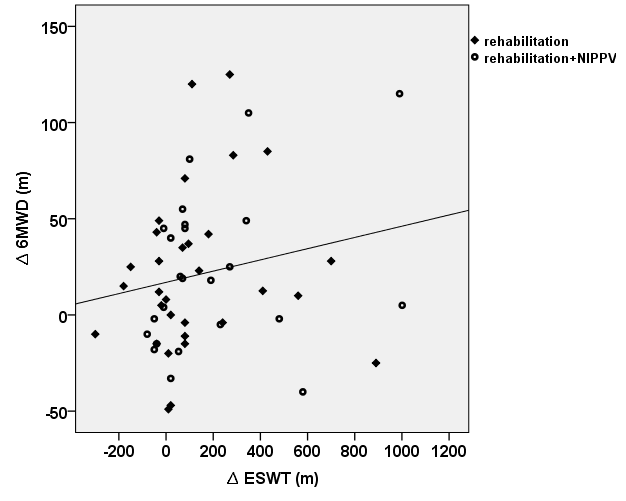

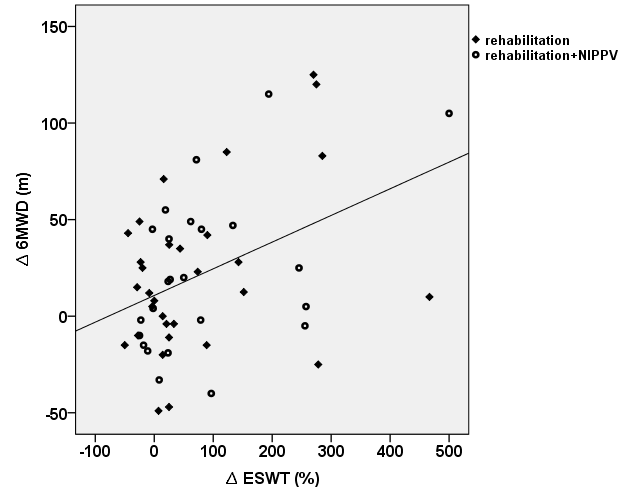

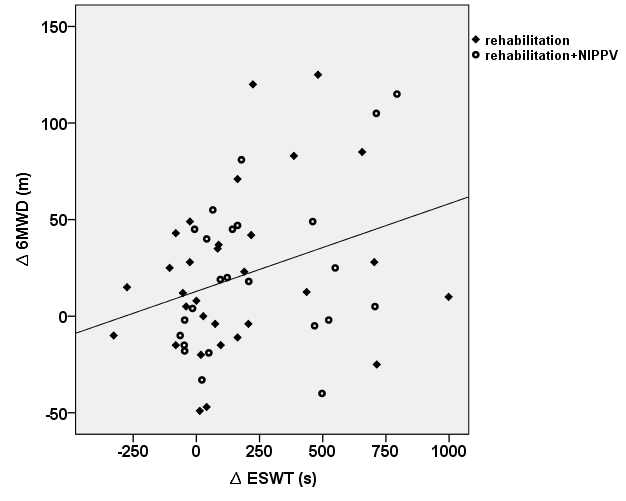

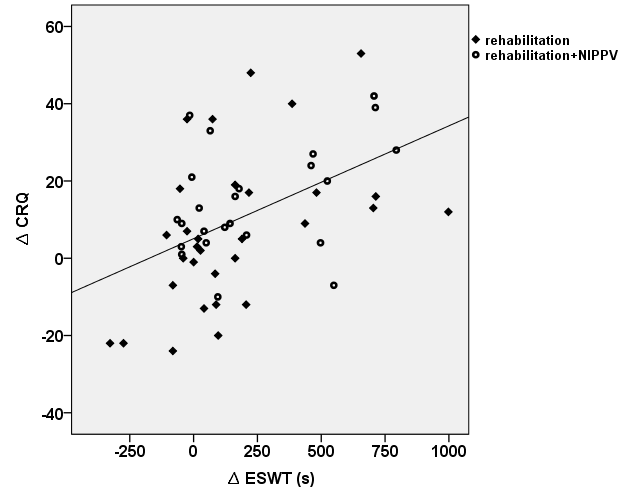

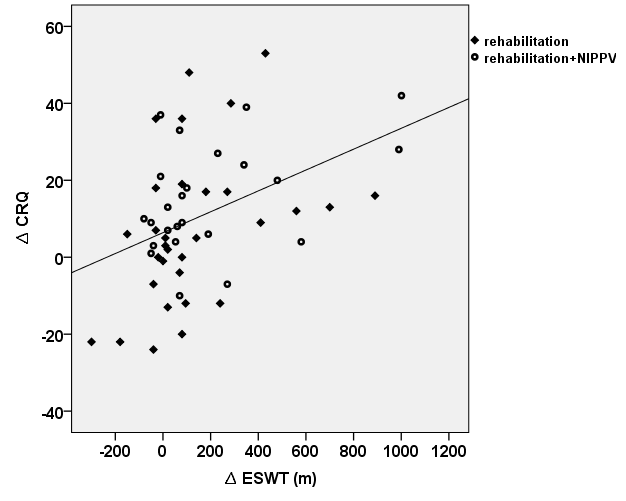

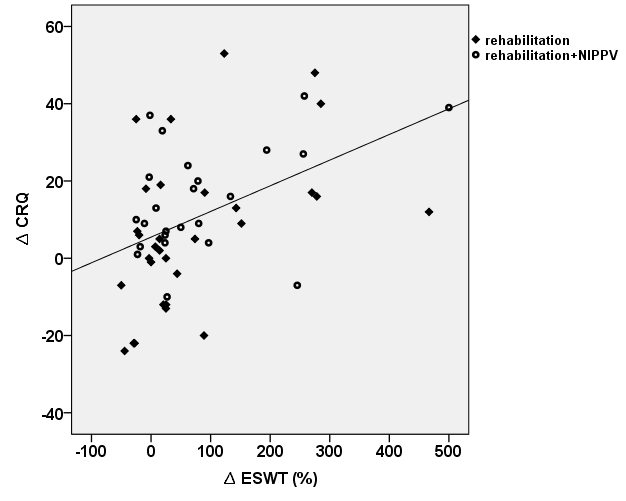

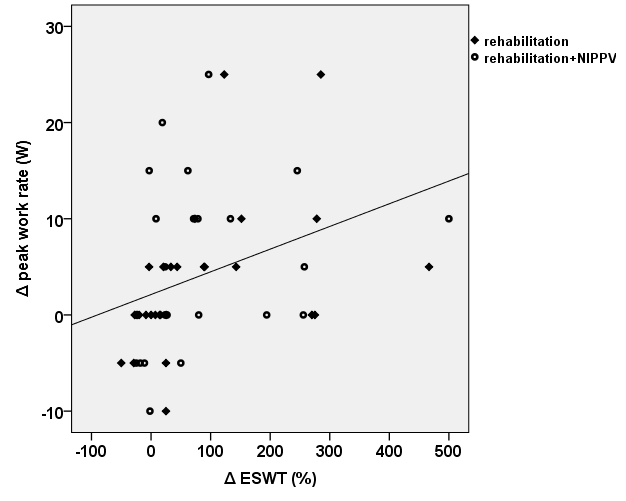

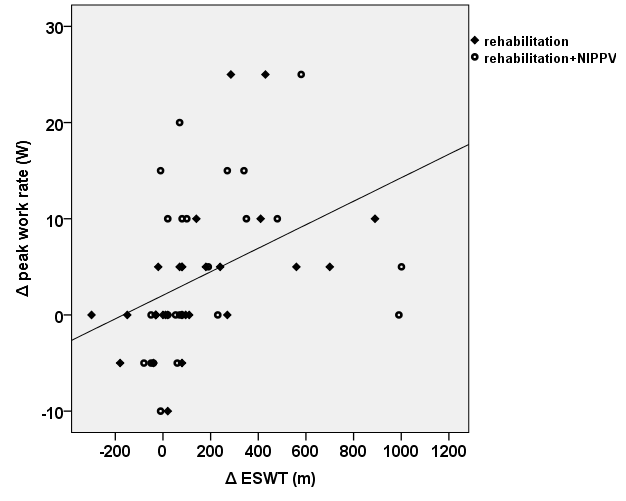

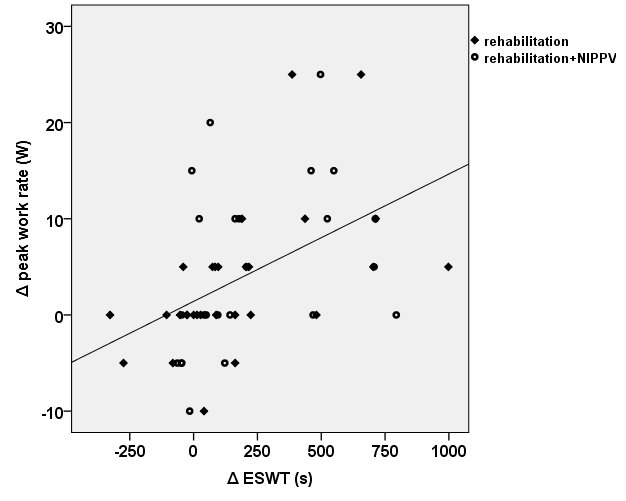

Supplement: Additional file 1: Figure S1. — a-i. Scatterplots of change in ESWT versus change in anchor variable. [file 12931_2015_182_MOESM1_ESM.docx]
