# Supplementary material for: Changes in the endurance shuttle walk test in COPD patients with chronic respiratory failure after pulmonary rehabilitation: the minimal important difference obtained with anchor- and distribution-based method
Source: Respir Res. 2015 Feb 19;16(1):27. doi: 10.1186/s12931-015-0182-x (PMC4336738; doi:10.1186/s12931-015-0182-x)
Supplement: Additional file 2: Table S1. — Regression equations used to calculate MID values. [file 12931_2015_182_MOESM2_ESM.docx]

| Table S1. Regression equations used to calculate MID values | | | | | |  |
| --- | --- | --- | --- | --- | --- | --- |
|  |  | Constant | B |  | 95% CI B |  |
| ΔESWT (s) | MID ESWT = | 111.47 + | 1.95 | * MID CRQ | 3.59-11.39 |  |
|  | MID ESWT = | 143.20 + | 2.24 | * MID 6MWD | 0.40-4.07 |  |
|  | MID ESWT = | 132.26 + | 16.66 | * MID peak work rate | 7.86-25.46 |  |
| ΔESWT (%) | MID ESWT = | 45.03 + | 3.09 | * MID CRQ | 1.42-4.76 |  |
|  | MID ESWT = | 51.38 + | 1.23 | * MID 6MWD | 0.49-1.98 |  |
|  | MID ESWT = | 59.84 + | 5.33 | * MID peak work rate | 1.37-9.29 |  |
| ΔESWT (m) | MID ESWT = | 91.30 + | 6.24 | * MID CRQ | 2.43-10.05 |  |
|  | MID ESWT = | 108.04 + | 13.9 | * MID peak work rate | 2.26-22.54 |  |
| ESWT: endurance shuttle walk test; MID: minimally important difference; CRQ: chronic respiratory questionnaire; 6WD: six-minute walking distance.  MID CRQ= 10 points, MID 6MWD= 25 meter, MID peak work rate= 4 Watt  The numbers in the equations are rounded off by 2 decimal places. For the calculation of the MID’s numbers were not rounded off (table 3). | | | | | |  |
|  |  |  |  |  |  |  |
|  |  |  |  |  |  |  |
|  |  |  |  |  |  |  |
